# Junctional adhesion molecule-C: A multifunctional mediator of cell adhesion

**DOI:** 10.1007/s00018-025-05829-z

**Published:** 2025-08-13

**Authors:** Klaus Ebnet, Michel Aurrand-Lions

**Affiliations:** 1 Institute-associated Research Group “Cell adhesion and cell polarity”, Institute of Medical Biochemistry, ZMBE, University of Muenster, Von-Esmarch-Str. 56, D-48149 Muenster, Germany; 2 Aix Marseille University, CNRS, INSERM, Institut Paoli-Calmettes, CRCM Equipe Labellisée, Ligue, Marseille, 2020 France

**Keywords:** Cell adhesion, Cell-Cell contacts, Cell Polarity, JAM-C, Junctional adhesion molecules, Migration, Tight junctions

## Abstract

Junctional Adhesion Molecule-C (JAM-C) is a member of the JAM family of cell adhesion molecules. JAM-C is expressed by a large variety of tissues including epithelial and endothelial tissues, neuronal tissues, glial cells, cells of the reproductive system, or cells of the hematopoietic system. Through trans-homophilic as well as trans-heterophilic interactions with the JAM family member JAM-B and with members of the integrin family JAM-C regulates diverse processes including epithelial barrier formation, leukocyte– endothelial interactions and transendothelial migration, neuronal cell migration along glial fibers, myelin sheath integrity, and germ cell migration. In this article, we review the biological activities of JAM-C, including its basic organization, its extracellular and intracellular interaction partners, and its diverse physiological functions.

## Introduction

Junctional adhesion molecules (JAMs) are members of the immunoglobulin superfamily (IgSF), a large family of proteins which are characterized by the presence of at least one Ig-like domains, the so-called Ig-fold [1]. The Ig-fold comprises about 100 amino acids (AA) which form a compact globular domain that consists of anti-parallel β-strands stabilised by a single disulphide bond [2]. The ancestral function of the Ig domain is to regulate cell-cell adhesion [1]. IgSF members exist from Caenorhabditis elegans to vertebrates [3], and the number of genes encoding IgSF proteins has strongly increased during the evolution from invertebrates to vertebrates [4]. The human genome contains more than 700 genes encoding IgSF proteins identifying the IgSF as the largest family and of secreted and cell surface-expressed proteins [5]. Also, the Ig domain is the most represented domain in human proteins [6]. Recent studies suggest that 445 members of the IgSF engage in at least 557 protein-protein interactions [7]. Given the number of Ig domain-containing proteins and the multitude of protein-protein interactions involving IgSF proteins it is not surprising that IgSF proteins regulate a multitude of processes during development and homeostasis.

Members of the JAM family include JAM-A, -B, and -C and are characterized by two Ig-like domains, a membrane-distal V-type Ig domain and a membrane-proximal C2-type Ig domain [1]. The JAM nomenclature has been adopted for other IgSF members with two Ig-like domains, including JAM4 [8] and JAML [9]. However, these proteins are more closer related to the IgSF members coxsackie- and adenovirus receptor (CAR), endothelial-cell-specific molecule (ESAM), and CAR-like membrane protein (CLMP), and form– besides the JAM-A, -B, -C subfamily– another subfamily of the CTX family [10, 11]. Interestingly, most members of the CAR subfamily including CAR, ESAM, CLMP, and JAM4 support cell aggregation after ectopic expression in heterologous CHO cells or L cell fibroblasts [8, 12–14]. A similar cell aggregation-supportive function has not been described for the JAM family members JAM-A, -B, and -C, which suggests that JAMs act predominantly in intercellular communication and signaling rather than in cell-cell cohesion. During the past years, a large variety of physiological functions have been identified for the three JAM family members ranging from the regulation of epithelial and endothelial cell-cell junctions, the development of the nervous system, the maturation of germ cells to the regulation of inflammatory responses [15]. The most extensively studied member of the JAM family, i.-e. JAM-A, has been the subject of several recent reviews [16–18]. Also, the roles of JAMs during cancer and inflammation have extensively been reviewed before [19–22]. In this review article we highlight the cell biological functions of JAM-C with an emphasis on its structural organization, its molecular interactors, and its role as mediator of cell-cell interactions in various contexts.

### Organization of JAM-C

**Fig. 1:**
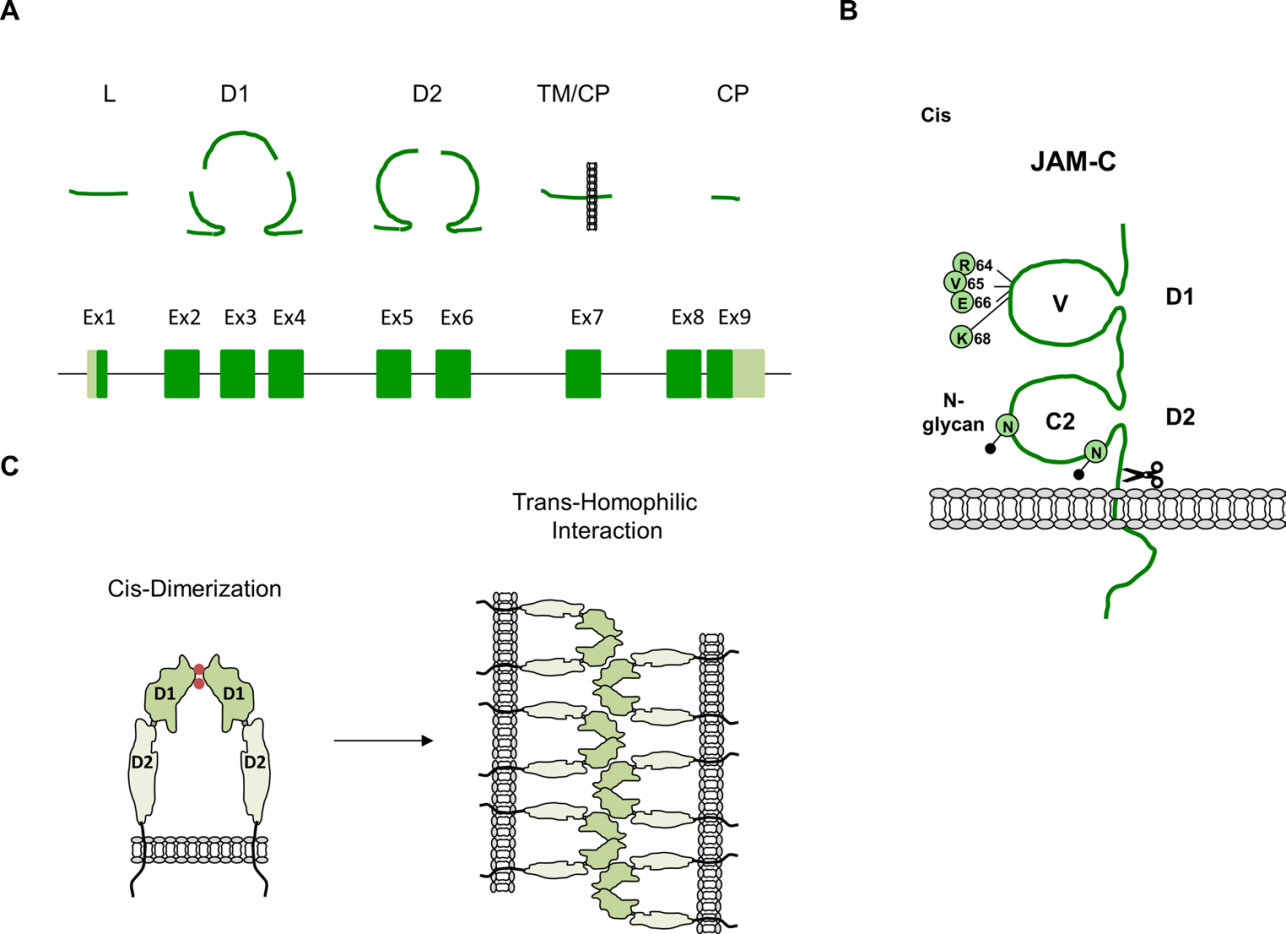
Principal organization of JAM-C.** A**: Genomic organization of the JAM-C gene. The JAM-C gene consists of nine exons. Coding regions are indicated in green, non-conding regions are depicted in lime-green. The JAM-C protein segments encoded by the coding regions of the nine exons are depicted in magenta. Abbreviations: L, leader peptide; D1, D1 Ig-like domain; D2, D2 Ig-like domain; ECD, extracellular domain; Ex, Exon; TM, transmembrane; CP, cytoplasmic. **B**: Organization of JAM-C. The two Ig-like domains are indicated by D1 (membrane-distal, V_35_ - S_127_, V-type) and D2 (membrane-proximal, P_139_ - D_236_, C2 type). Disulfide bridges involve C_53_– C_115_ (D1) and C_160_ - C_219_ (D2). Residues comprising the *cis*-dimerization motif (R_64_V_65_E_66_), a residue involved in trans-interaction with JAM-B (K_68_), and two potential N-glycosylation sites (N-glycan, N_104_, N_192_) are indicated by green circles. The scissor symbol indicates cleavage by ADAM10/17 transmembrane metalloproteinases. **C**: Cis-dimerization and trans-homophilic interaction of JAM-C. Cis-dimerization is most likely mediated by ionic and hydrophobic contacts involving the dimerization motif. Salt bridges between the two oppositely charged AA residues (E_66_•R_64_, R_64_•E_66_) are indicated by two rose-coloured dots. Cis-dimerization of JAM-C is predicted on the basis of a cis-dimerization motif that is conserved among JAM family members and that has been identified by X-ray crystallography of the JAM-A ectodomains (see text for details). Trans-homophilic interaction of JAM-C dimers results in JAM-C clustering at cell-cell contact sites

JAM-C has originally been identified through techniques like differential display or sequencing of expressed sequence tags (ESTs) [23–25]. Conflicting nomenclatures of murine JAM2 and human JAM3 molecules has led to a unifying nomenclature as JAM-C for this protein [26]. The gene encoding JAM-C contains nine exons (Fig. 1A). The mRNA encodes a protein of 310 amino acids (AA). The JAM-C protein consists of two extracellular Ig-like domains, a membrane-distal V-type Ig domain and a membrane-proximal C2-type Ig domain, a single transmembrane-spanning region, and a cytoplasmic region which consists of 48 amino acids (AA) (Fig. 1B). JAM-C can be cleaved by A Disintegrin And Metalloproteinase domain-containing protein (ADAM) 10 and ADAM17 resulting in a soluble form of JAM-C (sJAM-C) which consists of the two Ig-like domains [27, 28]. The first Ig-like domain contains a motif that consists of a hydrophobic AA flanked by two oppositely charged residue (R_64_-V_65_-E_66_ in human JAM-C, accession number Q9BX67, R_64_-I_65_-E_66_ in murine JAM-C, accession number Q9D8B7). An analogous motif (consensus: R[V, I,L]E) is present in JAM-A and JAM-B [15]. X-ray crystallography performed on JAM-A showed that this motif mediates cis-dimerization through two salt bridges formed between two oppositely charged AA on the two monomers (R_58_•E_60_, E_60_•R_58_) [29, 30], which suggests that cis-dimerization is conserved among all three JAM proteins. JAM-C also undergoes homophilic interactions in trans as evidenced by its exclusive localization at cell-cell contacts between two transfected cells [24, 31–33]. Mutating the Glu residue at position 66 of JAM-C to Arg (E_66_→R_66_) thereby preventing the two salt bridges between the monomers abolishes the trans-homophilic interaction [33]. These findings strongly support the notion that cis-dimerization followed by trans-homophilic binding regulates the enrichment of JAM-C at intercellular junctions (Fig. 1C). More recent studies identified K_68_ as an additional residue critical for the trans-interaction with JAM-B [34]. If this residue regulates exclusively the trans-heterophilic interaction with JAM-B remains to be determined.

### Intracellular interactors and posttranslational modifications of the cytoplasmic tail

**Fig. 2:**
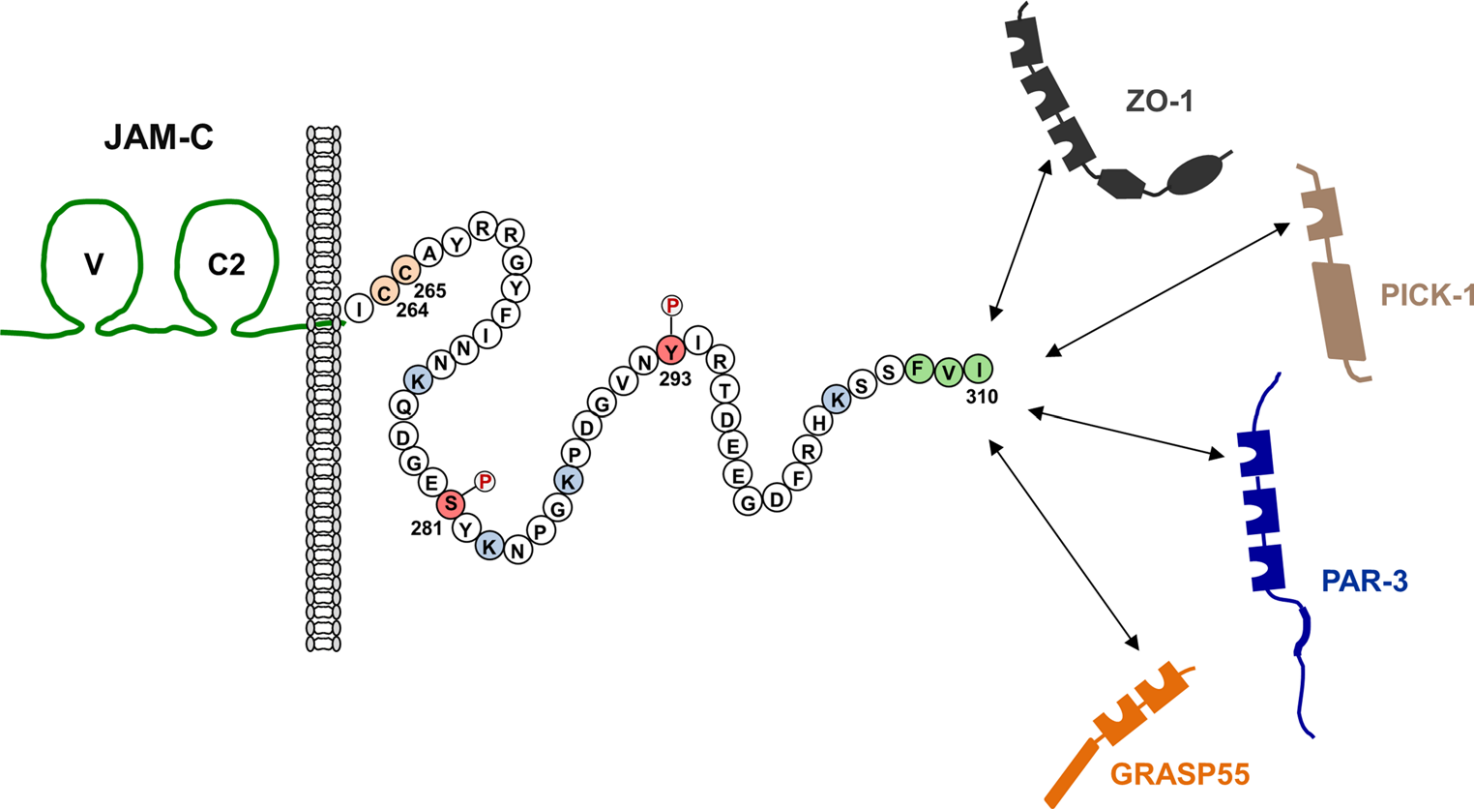
The cytoplasmic domain of JAM-C and posttanslational modifications. JAM-C contains a PDZ domain-binding motif (PBM, highlighted in green) at its C-terminus through which it directly interacts with the PDZ domain-containing scaffolding proteins ZO-1, PICK-1, PAR-3 and GRASP55. Two juxtamembrane cysteine residues which can be palmitoylated (C_264_, C_265_) are highlighted in rose. A serine residue and a tyrosine residue which can be phosphorylated (S_281_, Y_293_, modified by a encirceld P) are highlighted in red. Four lysine residues (K_276_, K_283_, K_287_, K_305_) which are potentially ubiquitylated are highlighted in blue. It is not clear yet if all or only some of the lysine residues are ubiquitylated

JAM-C interacts with several cytoplasmic proteins (Fig. 2). Direct interactors include Zonula occludens-1 (ZO-1) and Partitioning-defective 3 (PAR-3) [32], two PDZ domain-containing scaffolding proteins that are localized at tight junctions (TJ) of epithelial cells and endothelial cells [35, 36]. JAM-C also interacts with protein kinase C-alpha-binding protein (a.k.a. protein interacting with C kinase 1, PICK-1) [37], a scaffolding protein with a single PDZ domain and a BAR domain [38]. In addition, JAM-C directly interacts with Golgi reassembly-stacking protein 2 (a.k.a. Golgi reassembly-stacking protein of 55 kDa, GRASP55) [39], a protein that is implicated in the stacking of Golgi cisternae and in lateral linking of stacks within the Golgi [40]. In all four cases, the interaction is mediated through the PDZ domain-binding motif (PBM) of JAM-C and a PDZ domain of the scaffolding protein [32, 37, 39]. Based on validated yeast two-hybrid screening experiments a binary interaction of JAM-C with the transcription factor MEOX2 has been proposed [41]. This interaction, however, requires further validation by biochemical experiments.

The cytoplasmic region of JAM-C contains several residues that are post-translationally modified (Fig. 2). Two juxtramembrane Cys residues (C_264_, C_265_) are modified by S-palmitoylation mediated by the Zinc finger DHHC domain-containing protein 7 (ZDHHC7) palmitoyltransferase [42]. The addition of palmitic acid residues to the two cysteines of JAM-C regulates its efficient localization at cell-cell junctions [42]. In addition, the cytoplasmic region contains four lysine residues (K_276_, K_283_, K_287_, K_305_) that are putative targets of the E3 ubiquitin ligase Casitas B—lineage lymphoma (CBL) [43]. If all four residues or only some are ubiquitinylated remeins to be determined. Finally, two residues are subject to phosphorylation. Ser285 phosphorylation regulates the balance of cytoplasmic versus cell-cell junction localization of JAM-C in both CHO cells and KLN205 cells [32, 44]. A phosphoproteomic approach has identified Tyr293 as phosphorylation site [45]. The functional relevance of this phosphorylation is still unclear.

### Extracellular interactors

**Fig. 3:**
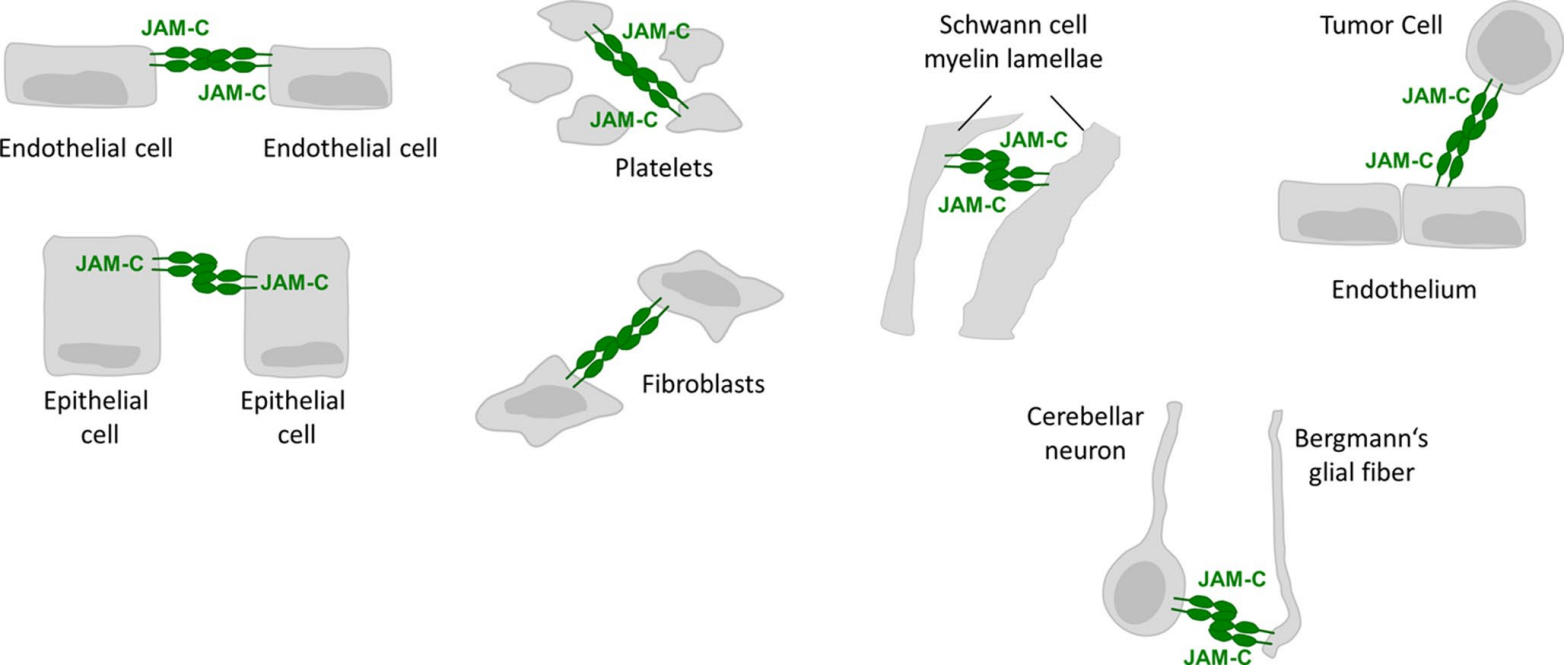
Homophilic interactions of JAM-C. JAM-C interacts with JAM-C in a trans-homophilic manner at various homotypic and heterotypic cell-cell contact sites. See text for details

**Fig. 4:**
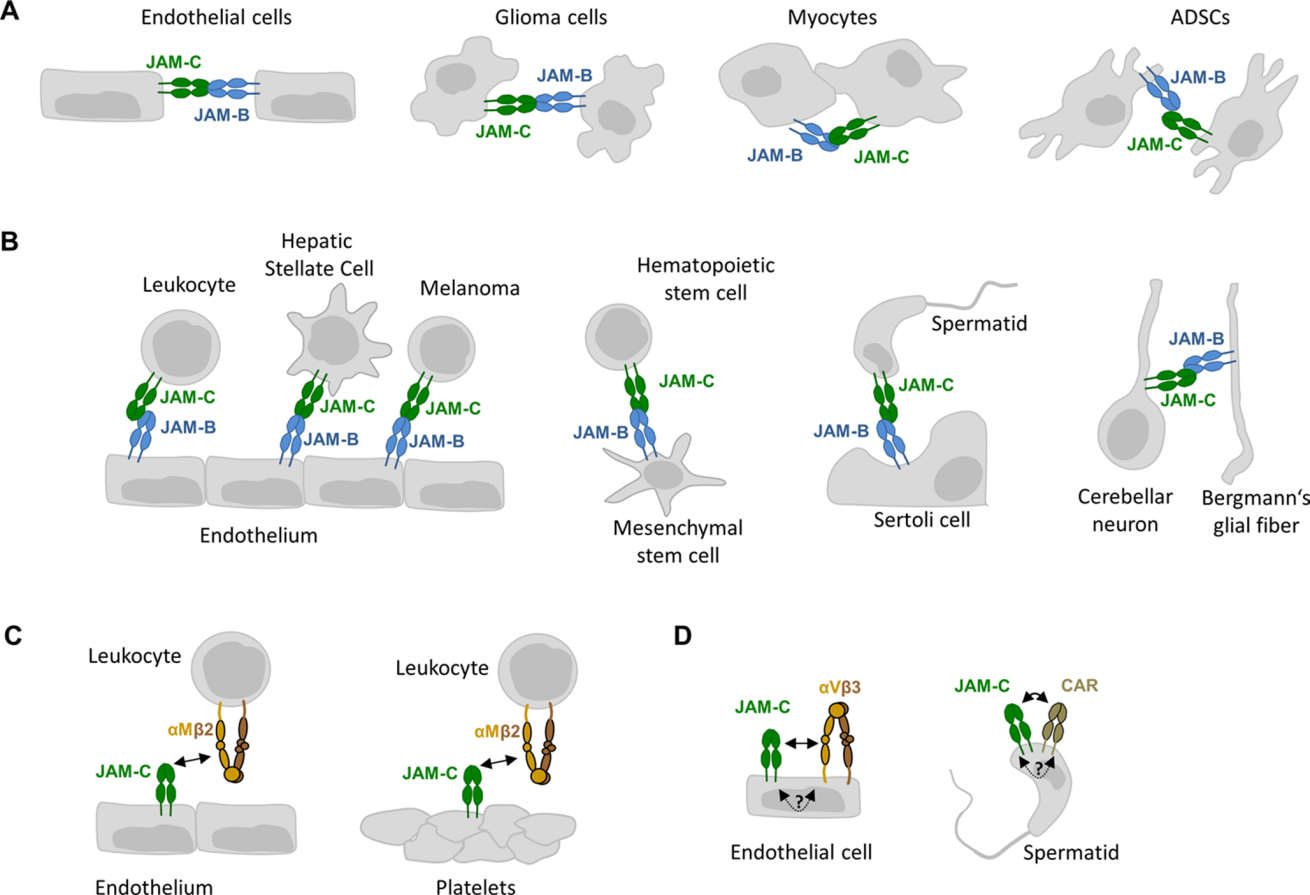
Heterophilic interactions of JAM-C.** A**: Heterophilic trans-interaction of JAM-C with JAM-B at homotypic cell-cell contacts. **B**: Heterophilic trans-interaction of JAM-C with JAM-B at heterotypic cell-cell contacts. **C**: Heterophilic trans-interaction of JAM-C with integrins at heterotypic cell-cell contacts. **D**: Heterophilic cis-interaction of JAM-C with integrins and IgSF members. Abbreviations: ADSC, adipose-derived stromal/stem cell; CAR, coxsackie- and adenovirus receptor. See text for details

Through its extracellular domain JAM-C interacts with various plasma membrane proteins on other cells including both other JAM family members as well as members of the integrin family (Figs. 3 and 4). As pointed out before, the extracellular domain of JAM-C interacts with JAM-C on.

adjacent cells through a trans-homophilic interaction. This interaction seems to be weak as experiments with recombinant JAM-C Fc fusion proteins suggest a low affinity interaction [25]. The JAM-C homophilic interaction, therefore, most likely does not significantly contribute to cell-cell adhesion but rather serves to localize and enrich JAM-C at specific sites of homotypic and heterotypic cell-cell contacts. Homophilic JAM-C– JAM-C interactions have been found at homotypic cell contacts between endothelial cells [24, 31, 46–48], epithelial cells [31, 49, 50], fibroblasts [51–53], and human platelets [54] (Fig. 3). In addition, JAM-C– JAM-C interactions occur at heterotypic cell junctions between endothelial cells and tumor cells [33, 55], and between neurons and glial cells in the brain [56], as well as at autotypic cell-cell contacts of Schwann cells in the peripheral nervous system [57, 58] (Fig. 3).

The JAM family member JAM-B is an important extracellular ligand of JAM-C [25, 59], and a number of cellular contexts in which a heterophilic JAM-C– JAM-B interactions is involved have been identified (Fig. 4A, B). Importantly, the heterophilic JAM-C– JAM-B interaction is stronger than the homophilic JAM-C– JAM-C interaction, as shown in experiments with recombinant proteins and by co-immunoprecipitation (Co-IP) experiments [25, 59], and as suggested by a more efficient recruitment of JAM-C to JAM-B-based cell junctions as compared to JAM-C-based cell junctions [59]. The higher affinity of the JAM-C– JAM-B interaction is likely to have implications on JAM-C– JAM-C-based adhesion complexes as the presence of JAM-B on interacting cells would be exprected to alter the stochiometry and/or the localization of JAM-C-based homophilic adhesion complexes. The JAM-C– JAM-B.

heterophilic interaction has been identified at homotypic as well as heterotypic cell-cell contact sites (Fig. 4). Homotypic contact sites involving JAM-C– JAM-B interaction include endothelial contacts [31, 32, 60], glioma cell contacts [61], and myocyte contacts [62] (Fig. 4A). Heterotypic contact sites involving JAM-C– JAM-B interaction include interactions of endothelial cells with leukocytes [63–65], fibroblasts [53, 66], and tumour cells [67] (Fig. 4B). In addition, heterotypic cell interactions involving JAM-C– JAM-B binding occur between hematopoietic stem cells and mesenchymal stem cells [68], between developing spermatids and Sertoli cells [69], and between cerebellar granular neurons and glial cells [56] (Fig. 4B).

Besides other JAM family members, JAM-C interacts with members of the integrin family. For example, JAM-C expressed by endothelial cells or platelets interacts in trans with αMβ2 integrin expressed by leukocytes to support leukocyte binding to endothelial cells and platelets during inflammation [59, 70–72] (Fig. 4C). JAM-C has also been found to be associated with αVβ3 integrin in endothelial cells [47]. It is likely that this interaction occurs in cis and is mediated by a member of the tetraspanin family since tetraspanin-mediated cis interactions with αVβ3 integrin, αVβ5 integrin and α3β1 integrin have been described for JAM-A [73–75] (Fig. 4D). Finally, in spermatozoa JAM-C exists in a complex with the JAM-related IgSF member Coxsackie- and Adenovirus Receptor (CAR) [76]. Since the molecules co-localize in the acrosome of isolated spermatozoa, this interaction also most likely occurs in cis (Fig. 4D).

### Cellular functions of JAM-C

Cell types expressing JAM-C include epithelial cells and endothelial cells, various leukocyte subsets and platelets, hematopoietic stem cells, vascular smooth muscle cells, cells of the nervous system including neural stem cells, neurons, Schwann cells and Müller glial cells, as well as cells of the male reproductive system, myocytes, and hepatic stellate cells. The JAM-C expression profile partially overlaps with the expression profiles of JAM-A and JAM-B (see ref [15] for a detailed representation of expression profils of JAM family members.) In fact, many cellular functions of JAM-C are mediated through its interaction with members of the JAM family.

#### Epithelial barrier formation

**Fig. 5:**
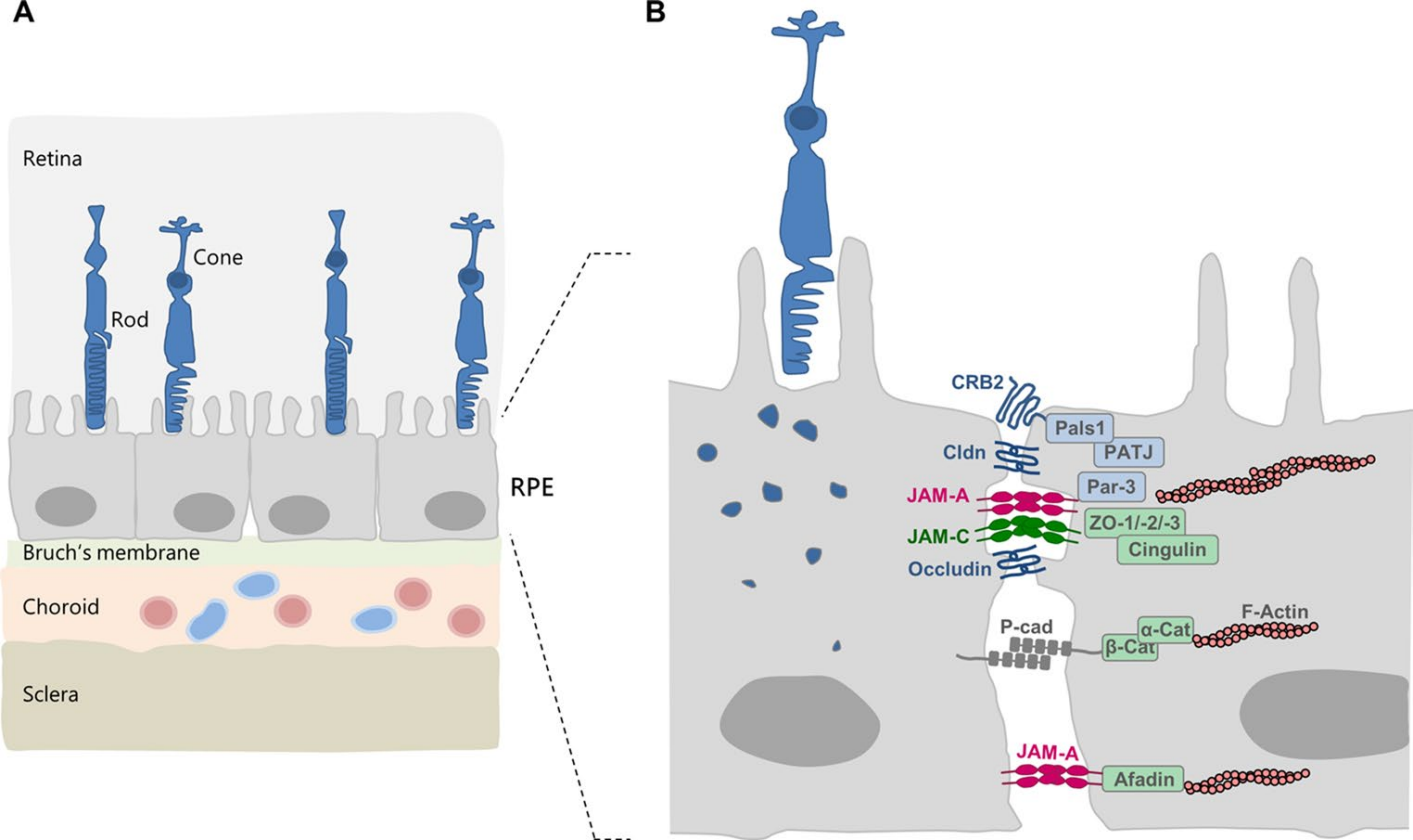
JAM-C localization in the retinal pigment epithelium.** A**: Anatomical localization of the retinal pigment epithelium (RPE) within the retina. The RPE is situated between the neural retina at the inside of the eye, and the choroid, a layer of connective tissue adjacent to the sclera at the outermost region of the eye. The Bruch’s membrane reflects the basement membrane formed by the RPE cells. The capillaries in the choroid (depicted as pink and light blue vessels) contain fenestrations to provide nutrients and to remove waste material from the RPE and the photoreceptors (rods, cones), which are in close proximity with the apical membrane of the RPE. Based on the presence of TJs the RPE cells form a barrier that prevents the free diffusion of macromolecules and ions across the paracellular space. **B**: RPE cells are highly polarized epithelial cells connected by tight junctions (TJs), adherens junctions (AJs) and desmosomes. The TJs are composed of integral membrane proteins typically localized at the TJs such as occludin, claudins, Crumbs2 (CRB2) and IgSF members including JAM-A and JAM-C, as well as scaffolding and adapter proteins such as zonula occludens (ZO) protein, Par-3, cingulin, PATJ and Pals1. The vesicular structures depict outer segments that were shed by adjacent cones and phagocytosed by the RPE cell

When ectopically expressed in MDCK cells, which represent a model cell line for polarized epithelial cells [77], JAM-C localizes to the most apical region of cell-cell junction suggesting a TJ localization and presumably TJ-specific localization [24, 31, 44]. In line with this notion, JAM-C directly interacts with the TJ-associated scaffolding proteins Par-3 and ZO-1 [32]. On the other hand, detailed studies on the expression and subcellular localization of endogenous JAM-C in polarized epithelial cells from different tissues are still missing. Among the best studied epithelial cell types regarding JAM-C expression are cells of the retinal pigment epithelium (RPE). RPE cells form a single layer of polarized epithelial cells which separate the neural retina from the underlying Bruch’s membrane and the choroid, a layer of connective tissue between the retina and the sclera that is rich in vasculature [78, 79] (Fig. 5).

One major function of the RPE is the transport of nutrients, ions, and water, but at the same time to form a barrier between the choroid and the photoreceptor cells, the so-called outer blood retina barrier (oBRB), which prevents unrestricted diffusion of solutes from the choroid to the sub-retinal space [79]. RPE cells contain typical TJs composed of various transmembrane components such as occludin, claudins, IgSF, and Crumbs2, as well as cytoplasmic scaffolding scaffolding proteins like ZO proteins and cingulin [78, 80].

JAM-C expression in RPE cells has been documented in several studies both in vitro and in vivo [49, 50, 81]. In primary human native RPE cells JAM-C co-localizes with the two TJ marker proteins occludin and ZO-1 but not with E-cadherin or desmoplakin, which are markers for AJs and desmosomes, respectively [49]. These findings indicate a localization of endogenous JAM-C at the TJs and suggest a role of JAM-C in TJ function. Of note, siRNA-mediated knockdown of JAM-C in RPE cells delays the localization of ZO-1 to primordial, punctate junctions and the recruitment of N-cadherin to mature intercellular junctions which suggests a function for JAM-C in the maturation and polarization process of epithelial cells [49], a function that has also been described for JAM-A [82].

**Fig. 6:**
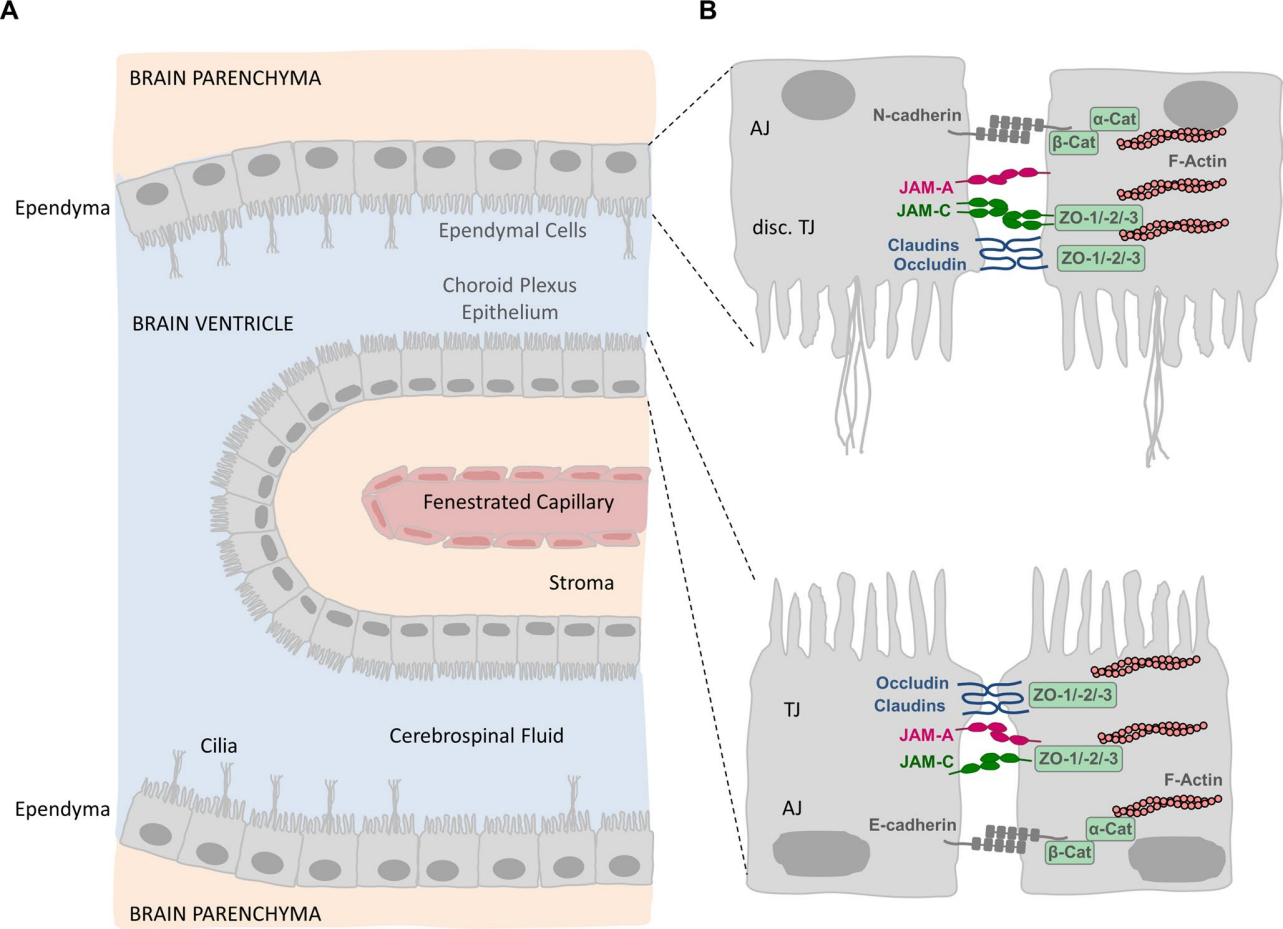
JAM-C localization in epithelia lining the brain ventricles: the choroid plexus epithelium and the ependyma. **A**: Anatomical localization of the choroid plexus epithelium and the ependyma. The choroid plexus is formed by fenestrated capillaries, the stroma, and the choroid plexus epithelium. The fenestrations of the capillaries allow for a rapid transport of water via the blood to the epithelial cells for the production and secretion of the cerebrospinal fluid (CSF). The CSF serves as a sink for nervous system waste and therefore is constantly secreted into and reabsorbed from the ventricular system. The secretion of the CSF into the brain ventricles is one major function of the choroid plexus epithelium. The ependymal cells separate the brain parenchyma from the brain ventricles. They are equipped with bundles of kinocilia that emanate form their apical surface. One major function of the ependymal cells is to generate and maintain a constant flow of the CSF through the ventricular system. **B**: Intercellular junctions of choroid plexus epithelial cells (bottom) and ependymal cells (top). Bottom panel: Choroid plexus epithelial cells are connected by barrier-forming TJs as well as AJs. The TJs restrict the free passage of solutes from the blood into the CSF, and vice versa. Top panel: Ependymal cells are connected by TJs and AJs. Their TJs are discontinuous and partially absent. Note that both JAM-A and JAM-C are localized at intercellular junctions of choroid plexus epithelial cells and ependymal cells. Abbreviations: AJ, adherens junctions; disc. TJ, discontinuous tight junctions

Epithelial cells other than RPE cells which express JAM-C include lens epithelial cells (LEC), i.e. epithelial cells that cover the anterior pole of the eye lens [83]. JAM-C co-localizes with ZO-1 at the apical surface in these cells. A lack of JAM-C results in smaller lenses, probably as a result of impaired LEC proliferation [83]. In addition, JAM-C is expressed by epithelial cells of the choroid plexus and by ependymal cells [61, 84, 85]. These two epithelial layers form a barrier between the blood and the cerebrospinal fluid (the blood-cerebrospinal fluid barrier, BCSFB), and between the cerebrospinal fluid and the brain parenchyma (the brain-CSF barrier), respectively [86, 87] (Fig. 6). As opposed to choroid plexus epithelial cells, ependymal cells form discontinuous TJs resulting in a partial barrier which nevertheless maintains and regulates the composition of the CSF [88]. Genetic deletion of the JAM-C gene results in hydrocephalus, possibly due to a defective barrier function or impaired polarization of the ependymal cells [85]. The precise mechanism, however, is not clear.

A selective breakdown of the barrier function in the ependyma but not in the choroid plexus epithelium has also been oberserved for the TJ-localized scaffolding protein MUPP1 [89], which is a cytoplasmic binding partner for JAM-A [90]. A more recent study identified JAM-C expression in airway epithelial cells [91]. Among the three major cell types present in the airway epithelium, i.e. multiciliated cells (MCC), basal cells, and secretory cells [92], JAM-C is specifically expressed in multiciliated cells in which it weakly localizes to cell-cell contacts but very prominently to EEA1-positive endosomes that are in close proximity to the apical membrane domain. Knockdown of JAM-C in cultured tracheal epithelial cells delays the epithelial barrier formation suggesting that JAM-C contributes to the formation of functional TJs [91]. The functional relevance of JAM-C enrichment in apical endosomes is still unclear.

#### Interendothelial junction formation and dynamics

JAM-C has originally been identified in vascular endothelial cells [24, 25], and expression of JAM-C by endothelial cells is documented by a large number of publications [31, 32, 43, 46–48, 55, 59, 66, 93–103]. Interestingly, in endothelilal cells derived from different vascular beds JAM-C is localized not only at cell-cell contacts but also in cytoplasmic stores from which it can be mobilized to interendothelial cell junctions by angiogenic growth factors like vascular endothelial growth factor (VEGF) and platelet-derived growth factor (PDGF) [46, 47, 95, 103]. Since angiogenesis is associated with dynamic cell rearrangements [104] the mobilization of JAM-C from intracellular stores by growth factors suggests that JAM-C contributes to the regulation of cell-cell interactions during processes that involve dynamic alterations of interendothelial junctions. In line with this assumption, a lack of JAM-C in microvascular endothelial cells prevents the increase in permeability that normally occurs in response to VEGF treatment, and stabilizes VE-cadherin-mediated adhesion between endothelial cells as a result of reduced actomyosin-based contractility [46]. Thus, one role of JAM-C in endothelial cells is to maintain junctional dynamics during processes that require reorganization of interendothelial junctions.

A more recent study strongly supports this notion. In cultured endothelial cells JAM-C is constantly re-internalized from the cell surface, in particular in response to stimuli which provoke junction reorganization [43]. Notably, JAM-C cotrafficks in cytoplasmic vesicles with proteins associated with endothelial permeability including VE-cadherin, neuropilin (NRP)−1 and − 2, and plasmalemma vesicle-associated protein (PLVAP) [105–107]. The recycling of JAM-C is regulated by E3 ubiquitin-protein ligase CBL which ubiquitylates JAM-C at several lysine residues resulting in its sorting to endosomes and multivesicular bodies, and subsequent degradation [43]. This mechanism allows for a rapid adaptation to situations that require changes in the integrity of interendothelial junctions, including collective cell migration. It will will be important to understand how the turnover of JAM-C contributes to the dynamics of junctional reorganization during processes like cell migration or leukocyte transendothelial migration.

An additional situation which requires a dynamic reorganization of endothelial cell-cell junctions is the development of a lumen to form a blood vessel. The process of lumen formation starts after migrating endothelial cells have engaged in cell-cell contacts through various cell adhesion receptors [108]. The recruitment of anti-adhesive, carbohydrate-decorated proteins to cell-cell contact sites results in the separation of the two membranes thereby generating a hollow space that eventually develops into a lumen [108, 109]. Depletion of JAM-C or its counter-receptor JAM-B blocks lumen formation in endothelial cells grown under three-dimensional culture conditions [110], a function that is probably related to the ability of JAM-C and JAM-B to interact with the polarity protein PAR-3 [32] and to activate the polarity regulator Cdc42 [110]. If JAM-C and JAM-B co-localize with other cell adhesion receptors such as VE-cadherin and N-cadherin at initial contact sites and move away durin lumen expansion to lateral sites, however, remains to be analyzed.

#### Neutrophil reverse migration

**Fig. 7:**
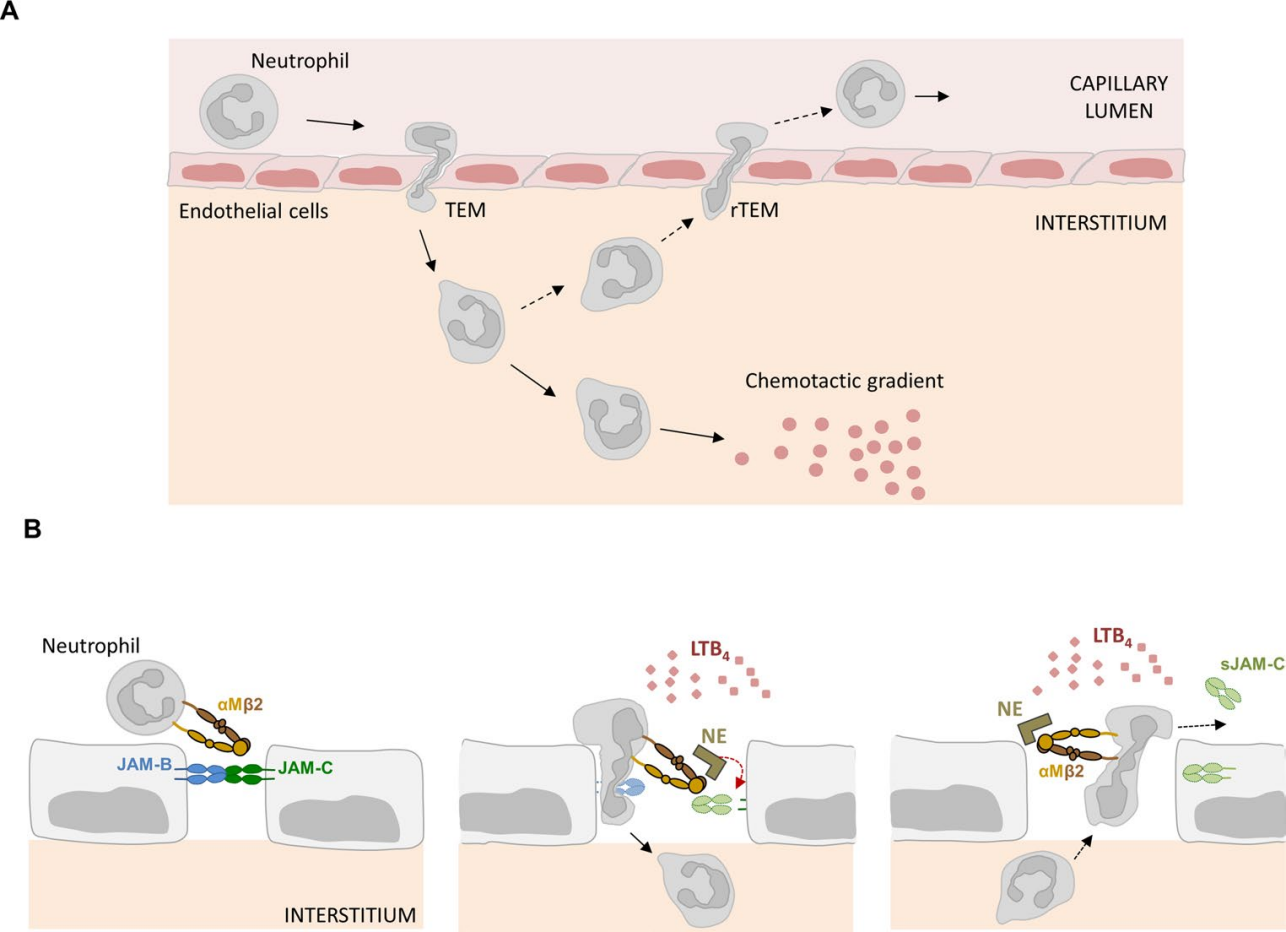
JAM-C regulates unidirectional transendothelial migration during inflammation.** A**: Neutrophil transendothelial migration (TEM) and reverse transendothelial migration (rTEM). At sites of inflammation neutrophils interact with endothelial cells, become activated and transmigrate through the endothelium by way of the paracellular pathway, i.e. along interendothelial cell junctions (transcellular TEM is not depicted here). After extravasation, neutrophils follow a gradient of chemokines to reach the site inflammation. In some cases, neutrophils do not follow the chemokine gradient but instead migrate towards the vessel wall and re-enter the blood circulation by transmigrating through the vessel wall in a ablumin-to-luminal direction (rTEM) resuling in the dissemination of activated neutrophils and possibly systemic inflammation. **B**: The JAM-C-based mechanism of neutrophil reverse transmigration. Left panel: During TEM, neutrophils interact with endothelial cell-expressed JAM-C through αMβ2 integrin. Pro-inflammatory Leukotriene B_4_ (LTB_4_) released by local inflammatory cells including neutrophils mobilizes surface localization of neutrophil elastase (NE) which is bound by αMβ2 integrin. NE cleaves JAM-C resulting in the destabilization of the JAM-B– JAM-C interaction and release of soluble JAM-C (sJAM-C) into the circulation. Right panel: Activated neutrophils present in the interstitial space cross the vessel wall in the reverse direction and re-enter the blood circulation. Note that JAM-C is also re-internalized during this process

Based on the expression by endothelial cells, leukocytes and platelets, and also based on the identification of the leukocyte integrins as trans-heterophilic interaction partners of JAMs, the contribution of JAMs to lymphocyte homing and tissue inflammation has been studied in great detail (outlined in the following review articles [15, 20, 111, 112]. During these processes, JAM-C seems to play a particular role in preventing that neutrophils that have migrated through the vascular wall into the interstitium re-enter the circulation by transmigrating through the endothelium in a abluminal-to-luminal direction, a process that is referred to as reverse transendothelial migration (rTEM) [113]. In the absence of functional JAM-C on endothelial cells, both monocytes and neutrophils show an increased rTEM activity [48, 114], JAM-C thus establishes a barrier at endothelial cell-cell junctions for transmigrated monocytes and neutrophils that prevents them from re-entering the circulation. For neutrophils, the underlying mechanism and pathophysiological relevance of this process has been further characterized. At sites of vascular injury, the lipid mediator leukotriene B_4_ (LTB_4_) released by various leukocyte subsets activates the αMβ2 integrin, a known ligand for JAM-C on neutrophils [70], and at the same time mobilizes neutrophil elastase which is released into the extracellular space and bound by αMβ2 integrin (Fig. 7). The direct trans-interaction of αMβ2 integrin with JAM-C enables αMβ2 integrin-bound elastase to proteolytically cleave JAM-C at endothelial cell junctions resulting in reduced JAM-C levels at EC junctions and a loss of the JAM-C-mediated gate function which regulates the transmigration of neutrophils in a polarized, i.e. luminal-to-abluminal manner. As a consequence, pro-inflammatory neutrophils migrate in an abluminal-to-luminal direction and are systemically distributed through the circulation, which eventually results in systemic inflammation [115, 116]. Observations of increased plasma levels of soluble JAM-C in various inflammatory conditions and in trauma patients underscores the pathophysiological relevance of this mechanism [27, 115, 117].

JAM-C, thus, is mandatory to build up a gate for the luminal-to-abluminal transendothelial migration. Although not entirely understood, this gate is probably established through its trans-heterophilic interaction with EC-expressed JAM-B, which forms a heterodimer with JAM-C. This heterodimer is stronger than the JAM-C homodimer resulting in an enrichment of JAM-C at EC contacts [59]. The interaction of JAM-C with monocyte- or neutrophil-expressed αMβ2 integrin may trigger signals that induce leukocyte polarization during transendothelial migration. In line with this notion, biophysical experiments combined with adhesion assays indicated that the mechanical strength of JAM-C– αMβ2 bonds is especially high for mediating neutrophil adhesion, polarization and crawling [72]. The absence or reduced expression of JAM-C at EC contacts at sites of injury or as a result of LTB4– NE signaling [100, 115] could possibly result in a lack of signals required for efficient leukocyte polarized migration.

#### Migration of differentiating granule neurons in the developing cerebellum

**Fig. 8:**
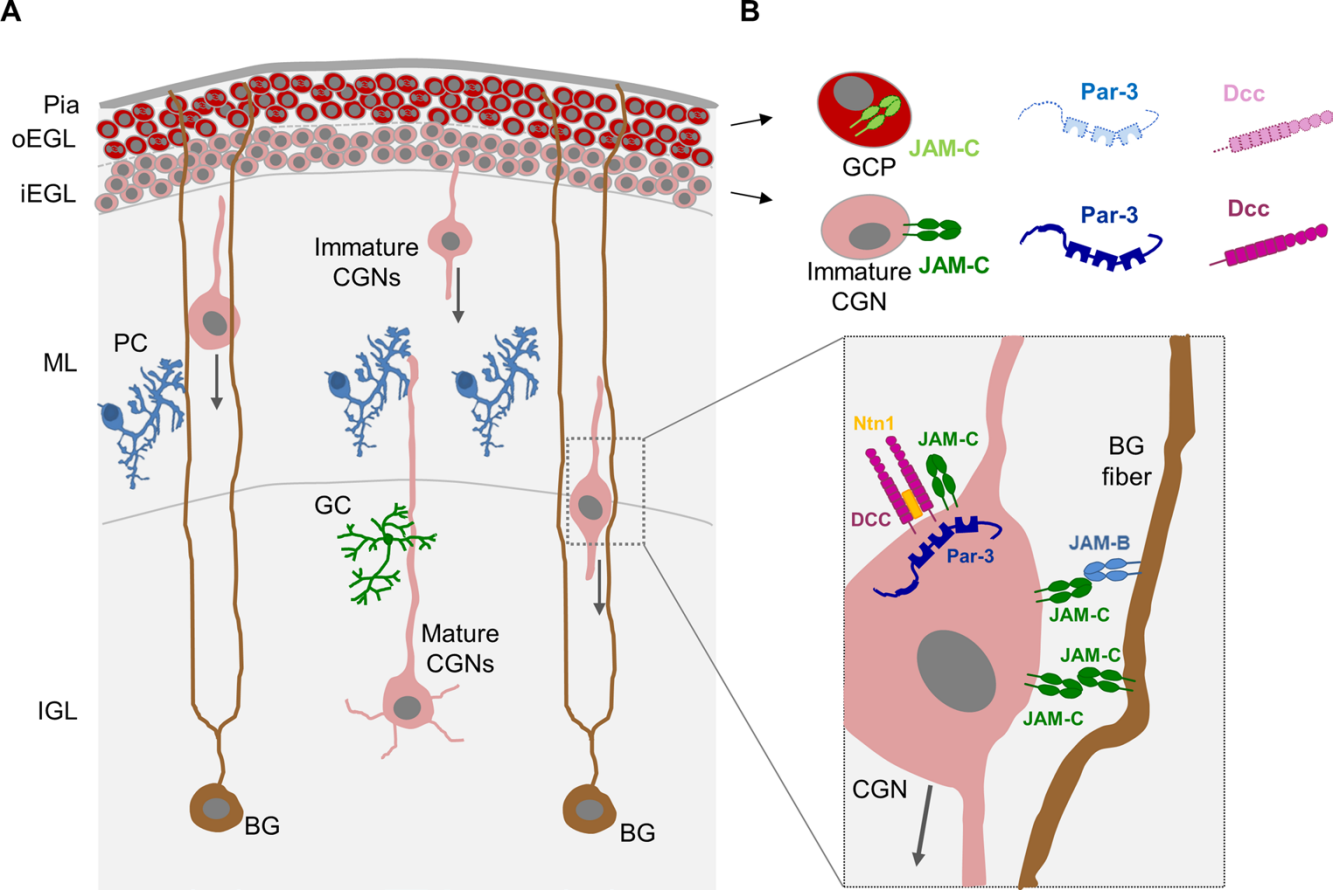
JAM-C in granule neurons of the cerebellum.** A**: Cerebellar granule neurons (CGN) derive from granule cell progenitors (GCP) which expand in the outer external granule layer (oEGL) by symmetric cell divisions. GCPs mature to newly differentiated CGNs which become postmitotic and form the inner external granule layer (iEGL). From the iEGL, newly differentiated CGNs extend radial protrusions and descend into the molecular layer (ML). During their radial migration immature CGNs use Bergmann’s glia cells (BG) as guide. At their final destination, i.e. the internal granule layer (IGL), the CGNs undergo final maturation by forming dendrites and synaptic connections with Golgi cells (GC), Purkinje cells (PC) and mossy fibers (not depicted). **B**: In GCPs present in the oEGL Par-3 expression is low due to degradation by the ubiquitin ligase Siah, and JAM-C is not at the cell surface. Postmitotic CGN present in the iEGL lose expression of Siah resulting in stabilization of Par-3 which regulates surface localization of JAM-C. Migrating CGNs use JAM-C to interact with and migrate along BG fibers through JAM-B and possibly JAM-C. The JAM-C– Par-3 complex also regulates surface localization of Deleted in colorectal cancer (DCC), a transmembrane receptor that mediates activities of the guidance cue netrin-1 (Ntn1), which acts as a repulsive cue to drive migration of differentiated CGNs away from the iEGL and the ML towards the IGL

In the developing cerebellum JAM-C has an important function in regulating the migration of immature neurons to the site of their final maturation. The cerebellum is composed of both excitatory and inhibitory neuronal cell types including cerebellar granule neurons (CGN, also called granule cells), Purkinje cells, Golgi cells, Stellate cells, and Basket cells [118]. Among these, the CGN are the most ubiquitous cell type making up more than 99% of all neurons [118]. During the development of the cerebellum, CGN arise from a precursor population present in a germinal zone, the externel granule layer (EGL), which is located close to the leptomeninges at the outermost region of the cerebellum [119]. At a certain stage of differentiation, the CGN become postmitotic and leave the EGL to migrate radially, i.e. inwards to reach their final destination, the inner granule layer (IGL). Here, they undergo final maturation, develop dendrites and form synaptic connections with Golgi cells and mossy fibers (Fig. 8). During inward migration, the cell respond to various signals including signals derived from cell-cell and cell-matrix interactions but also to soluble factors like growth factors and guidance molecules which can be attractive and repulsive [120].

Importantly, JAM-C seems to play a pivotal role in this process. JAM-C is absent in precursor cells present in the outer EGL but is surface exposed in cells present in the inner EGL [56] (Fig. 8). Surface expression is regulated by Par-3, a cytoplasmic interaction partner of JAM-C [32] (see also Fig. 2) that is constantly degraded through the actvity of the ubiquitin ligase Siah in cells present in the oEGL but stabilized in cells present in the iEGL where cells lose Siah expression [56]. Surface exposed JAM-C is now able to interact with Bergmann’s glia cells through hetero- and homophilic interactions with JAM-B and/or JAM-C (Fig. 8). JAM-C and Par-3 can also exist in a complex with Deleted in colorectal cancer (Dcc) [121], a transmembrane receptor for guidance cues such as Netrin-1 (Ntn1) which can mediate attraction or repulsion [122]. In the case of CGN, Ntn1 present in the iEGL and the ML acts a repulsive cue that contributes to the migration of differentiated CGNs away from the iEGL and the ML towards the IGL [121]. Through its molecular interaction with the polarity protein Par-3 and the guidance cue receptor Dcc JAM-C not only regulates migration along BG fibers but also contributes to the directionality of CGN migration.

Of note, the repulsive activity of Ntn1 operates only when cells migrate on vitronectin which is the predominant extracellular matrix component in the iGEL and the ML. JAM-C interacts with the vitronectin receptor αvβ3 integrin in endothelial cells [47] (Fig. 4). It is, thus, possible that JAM-C interact with αvβ3 integrin in GCN to regulate migration in the iGEL and the ML. A similar mechanism has been observed for JAM-A in MCF7 cells [74].

#### Myelination of peripheral nerves

**Fig. 9:**
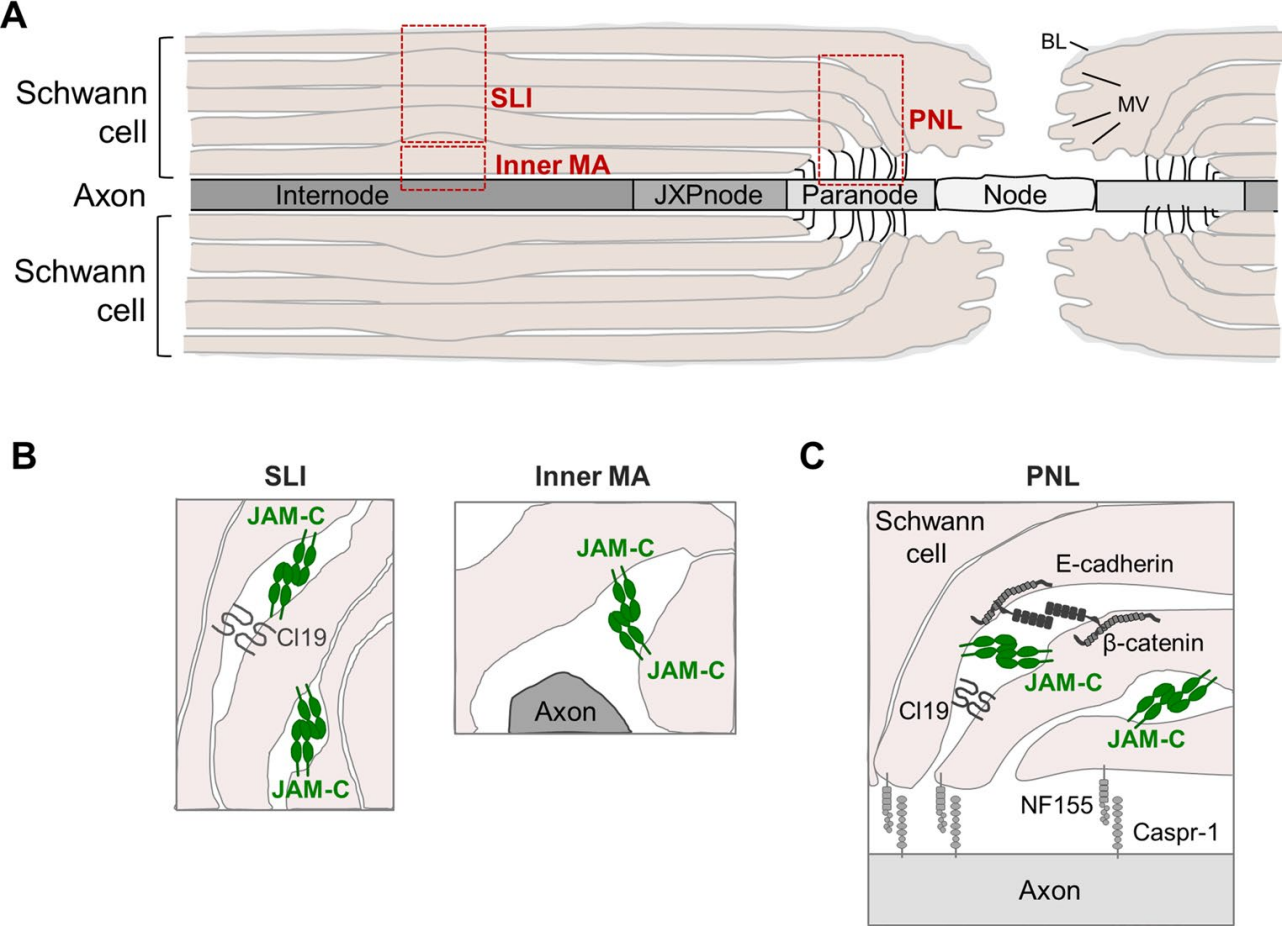
JAM-C at autotypic junctions of Schwann cells.** A**: Schematic longitudinal view of a myelinated axon in the peripheral nervous system. Myelinating Schwann cells at each side of the Node of Ranvier (Node) enwrap their lamellae (shown in rose) several times around the axon (shown in different grey values). The node of Ranvier represents a gap between two myelinating Schwann cells but is contacted by Schwann cell microvilli (MV). Areas of non-compact myelin with cytoplasmic spaces between apposing membranes (Schmidt-Lanterman incisures, inner mesaxon, paranodal loops are marked by red boxes. **B**: Cross-sectional view of two regions with non-compacted myelin, i.e. Schmidt-Lanterman incisures and inner mesaxon. **C**: Longitudinal view of a non-compact myelin region containing the paranodal loops. Note that JAM-C is localized exclusively in areas of non-compact myelin. Abbreviations: BL, basal lamina; Cl19, claudin-19; JXPnode, juxtaparanode; MA, mesaxon; MV, microvilli; PNL, paranodal loops; PNJ, paranodal junction; SLI, Schmidt-Lanterman incisure

A functional role of JAM-C in the peripheral nervous system has been identified in the course of experiments addressing JAM-C’s function in leukocyte transmigration. The studies revealed a specific localization of JAM-C in Schwann cells and its pivotal role in nerve conduction [57]. In the peripheral nervous system neuronal axons are in close contact with glial cells called Schwann cells. Schwann cells ensheath the axons with multiple layers of lamellae which ensure the rapid saltatory impuls propagation along nerve fibers. They also provide structural and metabolic support to the axons [123]. In most regions, the lamellae are closely apposed mediated by proteins like myelin basic protein (MBP) or proteolipid protein (PLP) leading to compact myelin. In some other areas, however, the lamellae are less closely apposed leaving some space between the lamellae resulting in loop- and channel-like structures filled with cytoplasm, the non-compact myelin. Non-compact myelin includes Schmidt-Lanterman incisures (SLI), the outer and inner mesaxon, and the paranodal loops (PNL) [124] (Fig. 9). A number of both integral membrane and cytoplasmic scaffolding proteins that are localized at epithelial and endothelial cell-cell contacts are localized at these areas of non-compact myelin [125].

JAM-C is expressed by Schwann cells where it is localized exclusively in areas of non-compact myelin including SLI, the inner mesaxon and the PNL [57, 58, 126–128]. Deletion of the JAM-C gene in mice results in structural alterations including misaligned paranodal loops, increased length of the nodes of Ranvier, and swelling of the myelin sheath. The lack of JAM-.

C also results in functional deficiencies like motor abnormalities and hypersensitivity to mechanical stimuli [57, 58]. Interestingly, JAM-C interacts through its extracellular domain with Peripheral myelin protein 22 (PMP22) when ectopically expressed in HEK293 cells [128]. PMP22 is primarily expressed in Schwann cells where it localizes to compact myelin but to a smaller extent also in non-compact myelin [129]. Loss of one allele of PMP22 in mice results in a mislocalization of JAM-C and in a phenotype that resembles an inherited neuropathy called hereditary with liability to pressure palsies (HNPP), which is also observed in JAM-C-deficient mice [57, 128]. In summary, the exclusive expression of JAM-C in Schwann cells suggest that the JAM-C-mediated adhesion of Schwann cell lamellae is necessary to maintain the structural organization of the myelin sheath and, thus, functional impuls propagation in the peripheral nervous system.

#### Differentiation of male germ cells

**Fig. 10:**
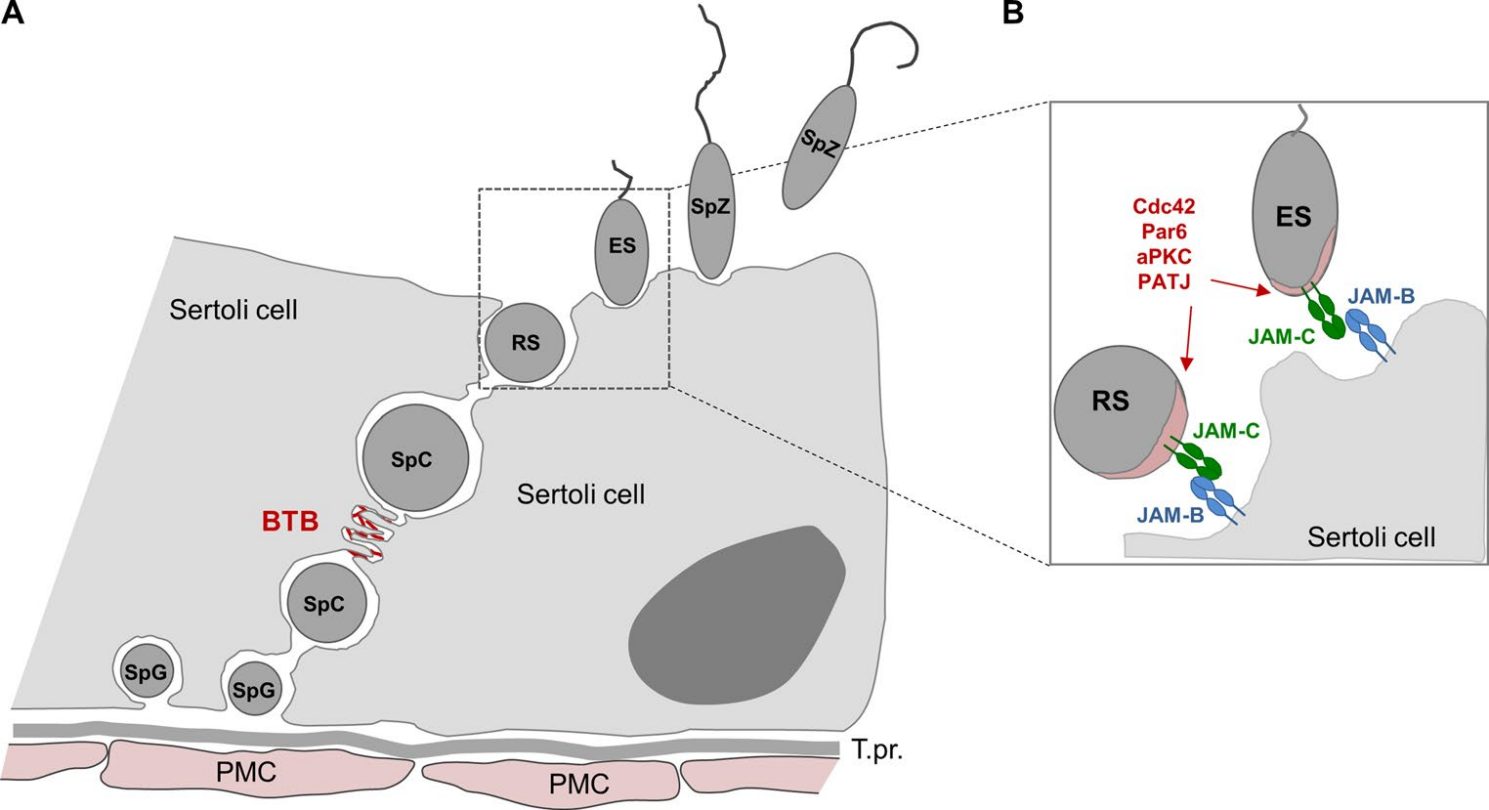
JAM-C in male germ cells.** A**: Schematic view of male germ cells developing in the seminiferous tubules. Spermatogonia (SpG) at the basal side of the seminiferous epithelium undergo meiotic DNA replication and differentiate into spermatocytes (SpC). Spermatocytes migrate across the blood-testis barrier and enter the adluminal compartment of the seminiferous tubules where they complete meiosis and differentiate into round spermatids (RS). Round spermatids differentiate further into elongated spermatids (ES) and fully mature spermatozoa (SpZ) which disconnect form the seminiferous epithelium by spermiation and are released in the lumen of the seminiferous tubules. During all stages of spermatogenesis the germ cells maintain intimate connections with Sertoli cells. **B**: JAM-C-mediated interaction between spermatids and Sertoli cells. Round and elongated spermatids express JAM-C to interact with Sertoli cells through JAM-B. Note that JAM-C recruits a Cdc42-Par6-aPKC polarity complex to the head structure of spermatids. In the absence of JAM-C, the Cdc42-Par6-aPKC polarity complex is mislocalized, acrosomal structures are absent, and further development of spermatids is arrested at the stage of round spermatids. Abbreviations: BTB, blood-testis-barrier; ES, elongated spermatid; PMC, peritubular myoid cell; RS, round spermatid; SpC, spermatocyte; SpG, spermatogonium; SpZ, spermatozoon; T.pr., Tunica propria

The deletion of the JAM-C gene in mice revealed an unexpected role of JAM-C in male fertility, a function that is most liklely mediated through its cytoplasmic association with polarity proteins such as PAR-3 and PAR-6 in spermatids [69]. Spermatids are generated from a small pool of spermatogonial stem cells which reside on the basement membrane that supports the seminiferous epithelium [130] (Fig. 10). These cells can self-renew to maintain a stem cell pool.

but also give rise to undifferentiated spermatogonia which differentiate into spermatocytes. Diploid spermatocytes enter meiosis giving rise to haploid spermatids which further differentiate form round spermatids to elongated spermatids and ultimately to spermatozoa that are released into the lumen of the seminiferous tubules. During all steps of this differentation process, the developing germ cells are in close physical contact with Sertoli cells, which represent a niche that provides soluble factors and membrane-bound signals to the developing germ cells [131, 132]. Also, the migration of germ cells from the basement membrane across the seminiferous epithelium to the adluminal compartment is a dynamic process that requires a constant remodeling of germ cell– Sertoli cells interactions. Many adhesion receptor systems found at epithelial cell-cell junctions are also found at Sertoli cell– Sertoli cell junctions including the blood-testis barrier (BTB) and at germ cell– Sertoli cell junctions [133–135].

During spermatogenesis JAM-C is expressed several types of spermatogenic cells including leptotene spermatocytes, round spermatids and elongated spermatids [39, 69, 76]. JAM-C is not expressed by Sertoli cells which, however, express JAM-B [69, 136]. In the absence of JAM-C the development of germ cells is arrested at the stage where round spermatids devlop into elongated spermatids (Fig. 10). As a consequence, the cells fail to develop into mature spermatozoa resulting in male infertility [69]. At the molecular level, the arrest at the stage of round spermatids correlates with the inability of JAM-C-deficient spermatids to recruit cell polarity proteins including Par6, PATJ, aPKCλ and Cdc42, which is most likely due to the inability to interact with Sertoli cell-expressed JAM-B. Par6, PATJ, aPKC and Cdc42 regulate the apico-basal polarization of epithelial cells but also the polarization of freely floating cells during their transient interactions with other cells [137, 138]. The interaction of germ cell-expressed JAM-C with Sertoli cell-expressed JAM-B, thus, promotes the morphological polarization of spermatids which is required for their functional maturation.

#### Hematopoiesis

Hematopoiesis occurs in the bone marrow (BM) of adult mammals and is the process by which mature blood cells are produced starting from Haematopoietic Stem Cells (HSC). HSC are subdivided in cells with long-term (LT-HSC) or short-term (ST-HSC) reconstitution potential, with the LT-HSC being considered as the most primitive quiescent stem cells. *Jam-C* expression by LT-HSC in adult mouse BM was first reported using highly purified cells and microarray analysis [139]. This was further confirmed using flow cytometry and with the functional demonstration that JAM-C-expressing haematopoietic cells are enriched for long-term multilineage reconstitution when transferred to lethally irradiated mice [140]. In line with this finding, mice deficient for *Jam-C* expression presented an imbalanced haematopoiesis with a significant myeloid-skewing. This was likely due to the loss of LT-HSC anchoring to the haematopoietic niche through JAM-C/JAM-B interaction since *Jam-B*-deficient mice phenocopy *Jam-C*-deficient mice [141]. Although this has been attributed to JAM-B expression by BM stromal cells interacting with LT-HSC, one could not exclude that JAM-C expressed within the BM microenvironment may also contribute to HSC maintenance as demonstrated with bioengineered niches containing recombinant soluble JAM-C [142]. JAM-C has also been shown to support human HSC adhesion to mesenchymal stromal cells and to be downregulated upon HSC mobilization in the periphery [68].

The observations that JAM-C is also expressed by human B lymphocytes, T lymphocytes, natural killer cells and dendritic cells [63–65] prompted several teams to test whether JAM-C may represent a biomarker for haematological malignancies. This was the case for acute myeloid leukemia (AML) in which JAM-C is expressed by a fraction of leukemic cells highly enriched for leukemic stem cells (LSC) and associated to a poor prognosis [143–146]. Such enrichment for LSC in the JAM-C-expressing fraction has been attributed to the maintenance of self-renewal through cis-interaction of JAM-C with LRP5 and downstream signalling to PDK1/AKT pathway [147]. In line with these studies, the conditional deletion of *Jam-C* before induction of AML using the iMLL-AF9 oncogene resulted in a shift from LT- to ST-HSC expansion, without affecting disease initiation and progression [148]. The shift in HSC expansion was correlated to a change in the transcriptional program that was also found in LSC from human AML patients. This suggests that JAM-C affects LSC transcriptional program through the control of the dynamic interaction between malignant haematopoietic cells and the BM microenvironment. A similar observation has been reported for multiple myeloma (MM), a haematological disease of antibody secreting cells that localizes primarily in the BM, but that can progress to a more disseminated disease [149]. Overexpression of JAM-C in a subset of MM cells was associated to CD138 downregulation and with specific localization of MM cells close to blood vessels in the BM [150]. Alltogether, these results indicate that JAM-C is involved in the dynamic localization of malignant haematopoietic cells within the bone marrow microenvironment.

### JAM-C signaling functions

Cell-cell adhesion receptors regulate a large variety of signaling pathways [151]. For the JAM family member JAM-A a function in transmitting and/or modulating cell-cell adhesion-triggered intracellular signal has been described in different contexts. For example, JAM-A regulates the activity of Rho and Ras family small GTPases [74, 152–154] and of c-Src [74, 155]. In addition, JAM-A stimulates various signaling pathways including the phosphatidy

inositol 3-kinase (PI(3)K)-Akt pathway [154, 156], the Hippo pathway [157] and the Erk1/2 signaling pathway [73, 74, 155, 158]. The function of JAM-C as stimulator of intracellular signaling pathways is less well explored, but recent evidence indicates a role in intracellular signaling as well.

A soluble JAM-B–Fc chimeric protein results in a rapid but transient c-Src activation [61] which suggests that ligand binding to JAM-C activates Src family kinases. Observations showing that the levels of active Src family kinases strongly correlate with the expression levels of JAM-C [143] further support this view. The signaling pathways have not been explored in detail. It is interesting that in contrast to JAM-C, JAM-A regulates c-Src in a negative manner [74, 155]. JAM-A exists in a complex with integrins and binds Csk, a negative regulator of c-Src, thereby inhibiting integrin-associated c-Src [74, 155]. These observations suggest that JAM-A and JAM-C regulate c-Src activity through different molecular mechanisms. The specific contribution to the activity status of c-Src, however, may also be context dependent.

Another important signaling function relates to the activation of Ras and Rho family small GTPases, a function of JAM-C which seems to be of particular importance in endothelial cells. Knockdown of JAM-C increases the activity of Rap1 and stabilizes endothelial intercellular junctions [46, 159], which indicates a negative regulatory function of JAM-C in Rap1 activity. Since Rap1 regulates both cadherin-mediated cell-cell adhesion and integrin-mediated cell-matrix adhesion [160], the role of JAM-C in regulating endothelial cell junctions, endothelial cell adhesion and endothelial sprouting [46] has been linked to the regulation of Rap1. It should be mentioned that a positive correlation of JAM-C expression levels and Rap1 activity in endothelial cells has also been observed [47]. This difference could be due to different vascular beds from which the endothelial cells were derived, i.e. microvascular endothelial cells derived from skin [46] and macrovascular endothelial cells derived from the umbilical vein [47]. In concert with JAM-B, JAM-C has also been found to regulate the activity of Cdc42 [110]. This observation was made in endothelial cells with double depletion of JAM-B and JAM-C leaving open the possibility that this activity is mediated by JAM-B. Based on experiments using siRNA-mediated knockdown of JAM-C or anti-JAM-C blocking antibodies, JAM-C also influences the Erk1/2 and p38 MAPK signaling pathways [50, 161]. In summary, JAM-C triggers several signaling pathways that are also regulated by JAM-A. In most cases, however, the underlying molecular mechanisms are less well explored compared to JAM-A, and further explorations are needed for a better understanding of the mechanisms underlying the role of JAM-C in signaling.

### JAM-C shedding

Similar to JAM-A [162], endothelial JAM-C can be cleaved by ADAM10 and ADAM17 metalloproteinases [27]. In addition, it can be cleaved by neutrophil elastase [115]. In both cases, the JAM-C ectodomain is shed from the endothelial cell surface resulting in a soluble form of JAM-C (sJAM-C) which consists of the two Ig-like domains. A biological activity of sJAM-C has been observed in the context of angiogenesis. When applied as a matrigel plug in mice or when added to three-dimensional endothelial cell cultures in vitro, sJAM-C promotes new blood vessel formation [27] indicating a proangiogenic activity. This proangiogenic activity of sJAM-C seems to be predominantly important during inflammatory processes, as a number of studies describe increased serum levels of sJAM-C in conditions that are associated with inflammation. These include systemic sclerosis [117], coronary artery stenosis [163], sepsis [164], age-related macula degeneration [103], and acute respiratory distress syndrome [115]. Since angiogenesis is often associated with inflammation [165], the release of sJAM-C under various inflammatory conditions most likely contributes to local new blood vessel formation.

Recent evidence suggests a possible additional biological function of sJAM-C. JAM-C and JAM-B are expressed by adipose-derived stromal/stem cells (ADSCs) [28]. These cells are distributed in the interstitial spaces between mature adipocytes and can differentiate into adipocytes and microvascular components [166]. In primary cultures of ADSCs as well as in mouse adipose tissue, a soluble form of JAM-C is detectable, and studies using the recombinant JAM-C ectodomain immobilized on tissue culture plates suggest that sJAM-C could promote adhesion and proliferation of ADSCs [28]. It is still unclar if sJAM-C released from ADSCs in the adipose tissue is immobilized at the extracellular matrix to stimulate adhesion and proliferation of ADSCs.

## Summary and conclusions

JAM-C is a cell adhesion receptor which is expressed in a variety of cell types. The present knowledge on the physiological functions of JAM-C implicate an important role in the development of epithelial and possibly endothelial barriers as suggested by its expression in barrier-forming epithelial cells such as proximal tubule kidney epithelial cells [167], RPE cells [49], choroid plexus epithelial cells and ependymal cells [85], and also suggested by its direct interaction with TJ-localized scaffolding proteins such as ZO-1 and Par-3. An analogous function in isolating cellular compartments is indicated by its expression in myelinating Schwann cells in which JAM-C localizes to sites of less compact myelin, i.e. paranodal loops, Schmidt-Lanterman incisures and outer and inner mesaxon [57]. Another important cellular role of JAM-C is regulatory function in cell migration which is coupled to developmental process in the central nervous system and in the male reproductive tract [56, 69, 121]. Interestingly, not all of the functions predicted by in vitro experiments have been confirmed by in vivo knockout model systems. In part, this might be explained by the co-expression of JAM-A, which shares several direct binding partners with JAM-C, i.e. the scaffoling proteins ZO-1, Par-3 and Pick-1, and which is co-expressed with JAM-C in several cell types [15]. Therefore, future studies using in vivo model systems with double JAM-A/-C deficiencies will be necessary to address putative compensatory mechanisms by JAM-A.

## Data Availability

N/A.

## Code availability

N/A.
